# MTMDAT-HADDOCK: High-throughput, protein complex structure modeling based on limited proteolysis and mass spectrometry

**DOI:** 10.1186/1472-6807-12-29

**Published:** 2012-11-15

**Authors:** Janosch Hennig, Sjoerd J de Vries, Klaus DM Hennig, Leah Randles, Kylie J Walters, Maria Sunnerhagen, Alexandre MJJ Bonvin

**Affiliations:** 1 Department of Physics, Chemistry, and Biology, Linköping University, SE-581 83 Linköping, Sweden; 2 Institute of Structural Biology, Helmholtz Zentrum München, Ingolstädter Landstr. 1, DE-85764 Neuherberg, Germany; 3 Department Chemie, Technische Universität München, Lichtenbergstr. 4, DE-85747 Garching, Germany; 4 Bijvoet Center for Biomolecular Research, Science Faculty, Utrecht University, 3584 CH Utrecht, The Netherlands; 5 Department of Informatics, Gabriele-von-Bülow Gymnasium, DE-13509 Berlin, Germany; 6 Department of Biochemistry, Molecular Biology, and Biophysics, University of Minnesota, Minneapolis, MN 55455, USA; 7 Biomolecular Dynamics, Department of Physics T38, Technical University München, DE-85747 Garching, Germany

## Abstract

**Background:**

MTMDAT is a program designed to facilitate analysis of mass spectrometry data of proteins and biomolecular complexes that are probed structurally by limited proteolysis. This approach can provide information about stable fragments of multidomain proteins, yield tertiary and quaternary structure data, and help determine the origin of stability changes at the amino acid residue level. Here, we introduce a pipeline between MTMDAT and HADDOCK, that facilitates protein-protein complex structure probing in a high-throughput and highly automated fashion.

**Results:**

A new feature of MTMDAT allows for the direct identification of residues that are involved in complex formation by comparing the mass spectra of bound and unbound proteins after proteolysis. If 3D structures of the unbound components are available, this data can be used to define restraints for data-driven docking to calculate a model of the complex. We describe here a new implementation of MTMDAT, which includes a pipeline to the data-driven docking program HADDOCK, thus streamlining the entire procedure. This addition, together with usability improvements in MTMDAT, enables high-throughput modeling of protein complexes from mass spectrometry data. The algorithm has been validated by using the protein-protein interaction between the ubiquitin-binding domain of proteasome component Rpn13 and ubiquitin. The resulting structural model, based on restraints extracted by MTMDAT from limited proteolysis and modeled by HADDOCK, was compared to the published NMR structure, which relied on twelve unambiguous intermolecular NOE interactions. The MTMDAT-HADDOCK structure was of similar quality to structures generated using only chemical shift perturbation data derived by NMR titration experiments.

**Conclusions:**

The new MTMDAT-HADDOCK pipeline enables direct high-throughput modeling of protein complexes from mass spectrometry data. MTMDAT-HADDOCK can be downloaded from http://www.ifm.liu.se/chemistry/molbiotech/maria_sunnerhagens_group/mtmdat/together with the manual and example files. The program is free for academic/non-commercial purposes.

## Background

It remains a major undertaking in the post-genome era to determine which biomolecules interact with each other, what function they have and to obtain their three dimensional high resolution structures. The main methods for achieving the latter are crystallography and nuclear magnetic resonance (NMR) spectroscopy, both of which can be time-consuming despite significant methodological advances. In addition, many targets elude high-resolution structural studies due to low solubility, low stability, large size or lack of crystal formation. Also, there is a rather limited number of structures of complexes compared to single proteins or domains thereof. There is thus a need for complementary methods that can give structural information on complexes since these are usually of higher interest from a biological point of view than single entities.

An alternative strategy to obtain structural information about biological macromolecular complexes is mass spectrometry. Here, the advantage is that sample requirements are low and the size limit is expandable to MDa complexes. Hydrogen exchange experiments coupled with mass spectrometry can yield very detailed information of protein folding and protein interactions, as reviewed in [1]. Recently, new methods used on large biological macromolecular complexes using ion mobility-mass spectrometry have been introduced, where gaseous ions are separated based on their size and shape [2,3]. Also, chemical cross-linking in combination with mass spectrometry can reveal structural insight into proteins and their interactions [4-8], while the employment of radical probe mass spectrometry (RP-MS, [9]) evaluated by PROXIMO can yield structural models of protein complexes [10]. These methods are, however, very sophisticated, requiring expensive state-of-the-art equipment and expert knowledge. In addition, time-consuming optimizations are often required, e.g. to find the right cross-linkers and conditions.

In contrast, limited proteolysis in conjunction with mass spectrometry (LP/MS) needs only a routine mass spectrometer and performing the experiments is straight-forward and fast. The resulting data can provide rather detailed information about protein interactions, stability and tertiary structure [11-17]. However, until recently, the extraction of this information was difficult due to the amount of data to be evaluated. MTMDAT, introduced in 2008 [18], is a tool for data processing, peak assignment, and visualization of mass spectrometry measurements, which greatly reduces the rate-limiting step of data evaluation and thereby enhances structural characterization of larger proteins and biomolecular complexes.

Here, we describe a novel implementation of MTMDAT, which streamlines the process from experimental work to an actual structural model of the complex. A new MTMDAT routine directly determines which residues are likely to be involved in a previously identified protein-protein interaction by comparing the mass spectra of bound and unbound proteins after proteolysis. In addition, a new pipeline between MTMDAT and HADDOCK [19-21] has been developed (see Additional file 1). HADDOCK is a docking method driven by (experimental) knowledge from a wide range of sources, e.g. mutagenesis, cross-linking or a variety of NMR experiments. Data-driven docking has the advantage that possible solutions are restricted a priori to be in agreement with experimental information. In HADDOCK, this is typically achieved through the definition of active and passive residues. Active residues correspond to interface residues identified by experiment, and passive residues are surrounding residues on the protein surface. HADDOCK enforces that every active residue is in contact with at least one active or passive residue on a partner molecule. When experimental information is sparse or absent, bioinformatic interface predictions can also be used [22,23]. HADDOCK allows for conformational change of the molecules during complex formation, and directly supports the docking of NMR structures containing multiple models. The coordinates of more than 90 biomolecular complexes solved using HADDOCK have been deposited in the PDB. The MTMDAT-HADDOCK pipeline allows the direct calculation of a three-dimensional model of the protein complex based on the interface residues identified by MTMDAT, provided that structure coordinates of the unbound components are available. This implementation, together with improvements in MTMDAT that increase its usability, enables direct high-throughput modeling of protein complexes from mass spectrometry data.

To demonstrate that the quality of the protein complex structures obtained from limited proteolysis/mass spectrometry data can be competitive compared to structures generated with more classical restraints from chemical shift perturbations (CSP) acquired by NMR, we studied the complex of the proteasome subunit Rpn13 with ubiquitin. Rpn13 is one of two known ubiquitin receptors in the proteasome [24,25] to which it docks via Rpn2/S1 [26-29]. In higher eukaryotes, it has an additional domain that contributes deubiquitinating enzyme Uch37 to the proteasome [26,27,30]. The structure of the Rpn13-ubiquitin complex has been solved by NMR spectroscopy using chemical shift perturbations upon complex formation and twelve unambiguous intermolecular NOEs ([PDB:2Z59]) [25]. In this study, this structure is used as control for validation of the MTMDAT-HADDOCK protocol.

Taken together, we present here an alternative approach that is quick and easy for obtaining restraints for data-driven docking. The results obtained by the presented LP/MS method are thus compared with data-driven docking using CSP alone as a method to obtain restraints for structure calculation and with purely bioinformatics driven docking using CPORT for interface predictions [31]. The resulting structure is comparable to that obtained by CSP experiments, and more accurate than CPORT bioinformatics interface predictions. The ease of performance, the gain in experiment time and the rapid and expert-free evaluation holds promise for LP/MS to contribute to the field of structural genomics.

## Results

The MTMDAT-HADDOCK workflow for obtaining the structure of the Rpn13-ubiquitin is described in the methods. In short, after performing the actual experimental work and acquiring the mass spectra, MTMDAT was used to get time-course plots and 3D plots (.csgnu files). This is described in the Methods section and in [18]. Rpn13 was readily digested and interaction restraints to ubiquitin could be derived from the cleavage pattern. For deriving ambiguous interaction restraints (AIRs) from these files, a threshold of 20% was used for Rpn13, meaning that the relative cleavage propensity of a given residue in the complex must be at least 20% smaller than for Rpn13 in the absence of ubiquitin. This high threshold was chosen to decrease the risk of false positives. Residues fulfilling this condition were chosen to be active residues for HADDOCK calculations. In contrast, ubiquitin had no susceptibility to proteases. For this reason, in the current study, the MTMDAT-derived active residues on Rpn13 were complemented by CPORT predictions providing passive residues on ubiquitin. Therefore, Rpn13-ubiquitin is also a test case for the ability of MTMDAT-HADDOCK to deal with missing LP/MS data, complementing them with predictions or data from other sources. For Rpn13, passive residues were chosen as described (close in space to active residues and with at least 50% solvent accessibility) and were automatically chosen by HADDOCK for all docking runs.

**Figure 1:**
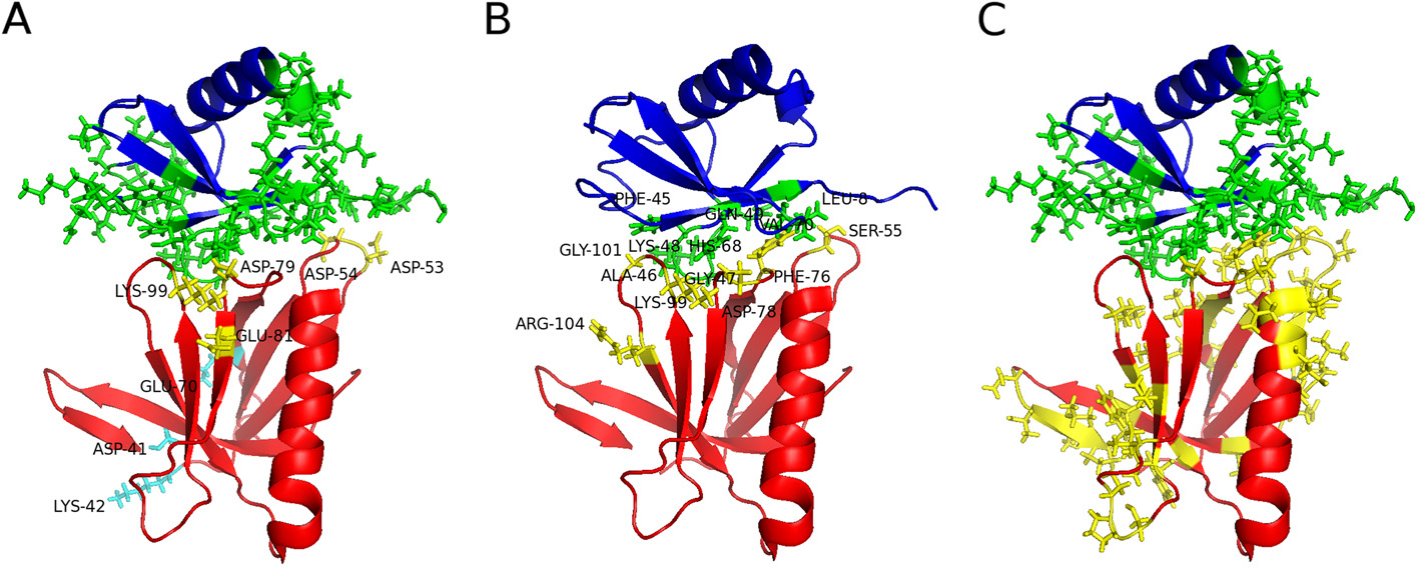
**Mapping of ambiguous interaction restraints.** Ubiquitin is coloured in blue and Rpn13 in red. Active residues are labeled and their sidechains are shown. Active residues for ubiquitin are colored in green and for Rpn13 in yellow. **(A)** Mapping of predicted ambiguous interaction restraints by limited proteolysis/mass spectrometry (ubiquitin interface residues are predicted from CPORT interface predictions). Residues in patch 1 are colored in light blue (D41, K42, E70), residues in patch 2 are colored in yellow (D53, D54, D79, K99). **(B)** Mapping of interface residues identified by NMR chemical shift perturbations, and **(C)** mapping of predicted interface residues by CPORT. This figure was generated with PyMOL [32].

In Figure 1, the active residues in protein-protein interaction identified by LP, CSP, and CPORT are mapped onto the published solution structure of the Rpn13-ubiquitin complex [PDB:2Z59], and Table 1 summarizes the active and passive residues that were used in all docking runs. Clearly, the limited proteolysis experiment produces two distinct patches (Figure 1A). The largest patch consists of four residues (D53, D54, D79, K99) that are indeed part of the interaction interface observed in [PDB:2Z59]. The other patch consists of three residues D41, K42, and E70, which are located on the opposite side. The program PATCHUP (see Additional file 1) has been developed and included in the pipeline, which automatically generates patches in an unbiased way (see Methods for further explanations). Chemical shift perturbations from NMR titration experiments [25] also give false positives. The amide resonance of R104 exhibits perturbations (Figure 1B), despite having no interactions with ubiquitin in [PDB:2Z59]. The generation of false positives is inherent to the LP/MS and CSP methods, due to possible conformational changes in regions further away from the binding interface upon complex formation, which result in chemical shift perturbations measured by NMR spectroscopy and could manifest in a higher accessibility to proteases assessed by the LP/MS method. Still, CPORT gives the largest number of false positives, predicting interacting residues on almost the entire surface of both proteins (Figure 1C).

**Table 1:** Active and passive AIRs chosen for the docking experiments

**Active and passive AIRs**		
**Docking Run**	**active AIRs**	**passive AIRs**
Baseline (BL)		
**Rpn13**	S55, L56, L73, I74,	auto
	F76, P77, D78, D79,	
	K99, A100, G101	
**ubiquitin**	K6, L8, I44, F45,	auto
	A46, G47, K48, Q49,	
	H68, V70, R72	
LP/MS filter (Ubiquitin CPORT)		
**Rpn13**	D41, K42, D53, D54,	auto
	E70, D79, E81, K99	
**ubiquitin**	-	F4, K6-K11, P19, Q31,
		E34-Q40, R42, I44-Q49, R54,
		T55, S57-N60, Q62-T66, H68,
		V70, L71-G76
LP/MS (Ubiquitin CPORT)		
**Rpn13**	D53, D54, S55, D78,	auto
	D79, F98, K99	
**ubiquitin**	-	F4, K6-K11, P19, Q31,
		E34-Q40, R42, I44-Q49, R54,
		T55, S57-N60, Q62-T66, H68,
		V70, L71-G76
chemical shift perturbations		
**Rpn13**	S55, F76, D78, K99,	auto
	G101, R104	
**ubiquitin**	L8, F45, A46, G47,	auto
	K48, Q49, H68, V70	
CPORT		
**Rpn13**	Y22-E25, R27, M31,	auto
	T36-P40, Q50-L56, H58,	
	V69, D71-I74,	
	F76, P77, D79, V85, C88-S90,	
	V93, V95, K103, L105, F106,	
	W108, E126, C127, N129, N130	
**ubiquitin**	F4, K6-K11, P19, Q31,	auto
	E34-Q40, R42, I44-Q49, R54,	
	T55, S57-N60, Q62-T66, H68,	
	V70, L71-G76	

In order to accommodate the presence of false positives and to choose the correct interaction surface in an unbiased way, a general docking calculation protocol has been developed. This protocol consists of two runs. The first run acts as a filter to choose the right patch if more than one is present. To this end, a random exclusion of 50% of AIRs for each structure calculation is performed. Since this increases the sampling space, a higher number of structures has to be calculated in each iteration (see Methods). In the case presented here, this filter was able to distinguish between false and true positives (Table 2). Here, the largest clusters, which also have the best HADDOCK scores have the correct patch 2 (yellow on Figure 1A) as the interface, as do clusters 5-8. Only cluster 3 and 4 have the wrong patch 1 (blue on Figure 1A) as the interface. Thus, it was possible to filter out the false positive (patch 1). However, there may be cases, where cluster size and HADDOCK score differ in ranking. In this case, the HADDOCK score is always prioritized. Subsequently, a second and final docking run was performed using only active residues from patch 2. For this second run two important changes have to be made. Residues whose side chains are surface exposed and are in immediate vicinity to active residues identified by limited proteolysis will also be included as active residues (S55, D78, and F98). This assumption is made since the limited proteolysis method can only sample a limited number of amino acids, which are cleaved by the proteases chosen. Therefore, surface neighbors of experimentally-identified residues, which would usually be defined as passive, are now also considered as active in the second run. Thirdly, no restraints are randomly excluded from the structure calculations. This exclusion is no longer necessary since the right patch should have been identified in the first filter docking run.

**Table 2:** Statistics of the LP/MS filter docking run

**Docking statistics of the filter run**				
**Cluster No.**^**a**^	**distance to patch 1 (Å)**^**b**^	**distance to patch 2 (Å)**	**HADDOCK score**^**c**^	**No. of structures**^**d**^
1	14.90	2.58	-80.0	44 (2)
2	11.39	3.47	-73.6	51 (1)
3	4.54	18.63	-73.4	24 (3)
4	7.45	15.01	-60.9	5 (8)
5	13.52	4.35	-60.5	6 (7)
6	23.12	3.57	-56.8	15 (5)
7	15.26	4.18	-50.4	15 (4)
8	19.99	4.08	-49.4	4 (9)
9	8.52	6.07	-46.5	9 (6)

^a^: Ranked by HADDOCK score.

^b^: The average distance of all residues in the interface of the docked model towards the center of the interface of the reference structure.

^c^: The average HADDOCK score of the four lowest energy structures in the cluster.

^d^: In parentheses the rank concerning the number of structure is shown.

The comparison between the three docking calculations, LP/MS, CSP, and CPORT are shown in Table 3. The performance of our MTMDAT-HADDOCK protocol was assessed as follows (in decreasing importance): i) low average RMSD values for the superimposition of the 10 lowest energy structures of the cluster with the best HADDOCK score onto [PDB:2Z59] (here, assessment according to CAPRI was used, where a model is assessed to be acceptable, if the iRMSD is below 4 Å, the lRMSD below 10 Å, and the fraction of native contacts (fnat) larger than 0.1 [33], ii) HADDOCK score of the four lowest energy structures (at the interface) in the cluster closest to the target structure [PDB:2Z59], iii) rank of the cluster with the best structure (closest to reference [PDB:2Z59]) based on the HADDOCK score, and iv) number of structures in the cluster closest to [PDB:2Z59] (if HADDOCK scores are within the standard deviations).

**Table 3:** Statistics of docking calculations

**Docking statistics of Rpn13-Ubiquitin**						
**Parameter**	**BL**^**a**^	**LP/MS**	**LP(all)**^**b**^	**CSP-50**	**CSP-0**	**CPORT**
Random excl.	yes, 50	no	no	yes,50	no	yes, 87.5
No. of clusters	4	9	7	9	2	25
Best cluster ^c^	1	1	2	4	1	5
No. of structures	333	141	79	11	258	13
iRMSD to 2Z59 ^d^	11.24	2.85	11.51	6.70	10.13	7.99
lRMSD to 2Z59 ^e^	21.19	7.76	19.97	13.52	19.11	16.63
fnat ^f^	0.044	0.124	0.012	0.018	0.053	0.003
HADDOCK score ^g^	-78.6	-65.8	-77.5	-72.0	-71.2	-75.4
**Best cluster to 2Z59**^h^	3(2)	1(1)	4(2)	3(2)	2(2)	9(4)
No. of structures	45	141	95	116	141	14
Best iRMSD to 2Z59 ^i^	2.77	2.71	2.66	2.86	1.80	5.00
Average iRMSD to 2Z59	3.79	2.85	3.06	3.23	3.41	5.08
Best lRMSD to 2Z59	6.56	6.42	6.67	7.85	7.99	9.65
Average lRMSD to 2Z59	8.60	7.76	7.07	8.63	9.71	11.12
Best fnat	0.276	0.155	0.147	0.232	0.351	0.008
Average fnat	0.153	0.124	0.090	0.182	0.237	0.003
HADDOCK score	-52.8	-65.8	-60.4	-63.2	-58.3	-40.6

^a^: BL is a baseline run, where all contacts from the reference structure 2Z59 were included as active residues.

^b^: LP(all) is similar to the LP/MS run, but not using CPORT prediction for ubiquitin, but instead all 76 ubiquitin residues as passive residues.

^c^: The cluster with the lowest average HADDOCK score. The number refers to the rank in cluster size.

^d^: iRMSD is the interface RMSD (within 10 Å of the interface), between the average structure in the cluster and the lowest energy structure of the reference [PDB:2Z59].

^e^: lRMSD is the ligand RMSD (the average structure and lowest energy structure of the reference ensemble [PDB:2Z59] are fitted on the backbone atoms of the larger protein, Rpn13, and the RMSD is calculated for the smaller, ubiquitin), between the average structure in the cluster and the reference [PDB:2Z59].

^f^: The fraction of native contacts is calculated by counting all contacts (residue-wise) between Rpn13 and ubiquitin in the reference structure and comparing how many are retrieved for the average structure in each cluster.

^g^: The average HADDOCK score of the four lowest energy structures in the cluster (in kcal/mol).

^h^: Deemed by the lowest iRMSD compared with [PDB:2Z59] of the four lowest energy structures in the cluster. The first number is the rank in HADDOCK score, and the second number in parentheses is the rank in cluster size.

^i^: The “Best” values are the comparison between the one best structure in each cluster and the reference, whereas the “Average” values are averaged over the ten lowest energy structures of each cluster.

**Figure 2:**
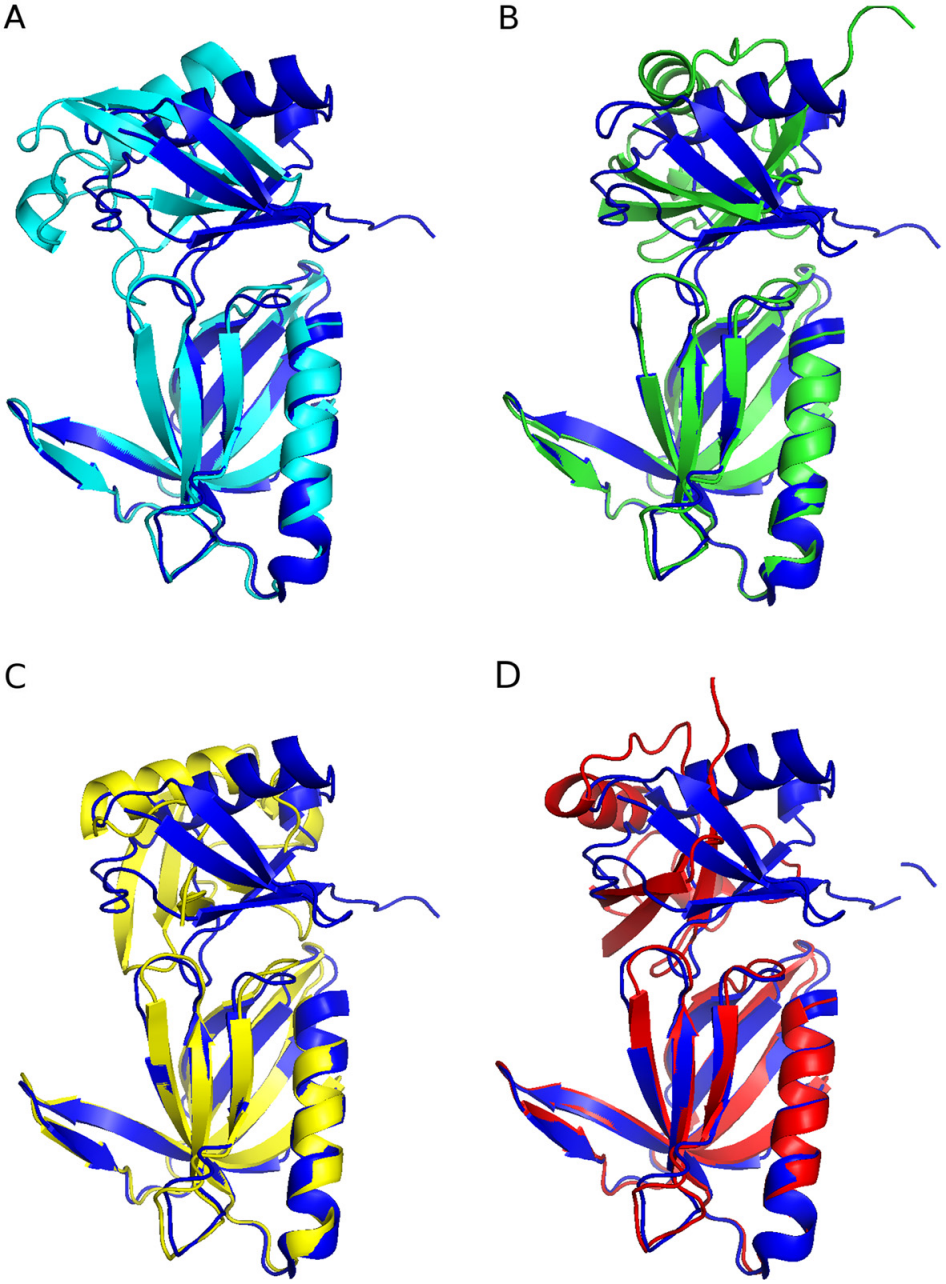
**Best structures of the docked Rpn13-ubiquitin complex structures compared with the high resolution structure.** The best structures, concerning their RMSD at the interface compared with [PDB:2Z59] of all methods are overlayed onto [PDB:2Z59] (dark blue). Structures based on limited proteolysis are colored in light blue, based on chemical shift perturbations in green (CSP-50) and yellow (CSP-0), and based on CPORT predictions are colored in red. **(A)** Comparison of LP/MS and with [PDB:2Z59] (2.85 Å to target). **(B)** Comparison of CSP-50 with [PDB:2Z59] (6.70 Å to target). **(C)** Comparison of CSP-0 with [PDB:2Z59] (10.13 Å to target). **(D)** Comparison of CPORT with [PDB:2Z59] (7.99 Å to target). This figure was generated with PyMOL [32].

In all four categories, the MTMDAT-HADDOCK method performed better than the CSP and CPORT approach. CPORT performed worse, since it has to sample many possible patches on the entire surface of the protein. The user, who has in applied cases no knowledge about the right target structure, would have chosen the right cluster in the LP/MS method, since the largest cluster with the best HADDOCK score contains structures closer than 3 Å to the target structure. For the experimental CSP data two different runs were performed, with and without random removal of AIRs. For both runs, the correct structure was generated, but with an RMSD slightly worse than the LP/MS run (Table 3). More importantly, in both runs, the correct structure was not ranked as the top cluster, and the top cluster corresponds to a structure that has a large backbone RMSD to the target structure. From Figure 2, where the lowest energy structure of the top ranked clusters is superimposed on the target structure, it becomes apparent that, if the top HADDOCK cluster would have been selected, a structure close to the target structure would have been only obtained with the LP/MS method. Clearly, CSP without random removal of AIRs performs worst (backbone RMSD of 10.13 Å), followed by CPORT (7.99 Å), and CSP with 50% random removal of AIRs (6.70 Å). The LP/MS method comes closest with a backbone RMSD of 2.85 Å. CSP-50 has the same set up as the filter docking run applied to the LP/MS data. Here, the false positive R104 would not have been filtered out, since the cluster with the best HADDOCK score is with 6.70 Å far away from the target structure, and the cluster closest to the target is ranked only third in HADDOCK score and second in cluster size (Table 3).

The Rpn13-Ub complex is a difficult docking case, in the sense that HADDOCK has difficulties in reproducing the experimental structure of the complex. As a positive control, the true interface (i.e. all interface residues from [PDB:2Z59], Table 1) was selected as active residues. However, even for this baseline run with perfect interface data (Table 3, BL), only the third-ranked HADDOCK cluster corresponds to [PDB:2Z59] (Table 3, BL run). Moreover, random removal of residues was necessary to get even this result: an alternative baseline run without random removal failed (results not shown). Also, the baseline run structures were no more accurate than the LP structures (best iRMSD of around 2.7 Å). Moreover, we repeated both runs without using passive residues that only the true interface is used in docking calculations (data not shown). The run with random removal showed similar results, but with worse iRMSD (average: 3.84 Å, best: 3.24 Å). The run, where no active residues were removed slightly improved. A second cluster (second-ranked in HADDOCK score) with an average iRMSD to 2Z59 of 4.0 Å (best: 3.81 Å) appeared. Still, this is a worse performance than the LP run.

Finally, we tested the dependency of the LP method on CPORT in the case of Rpn13-Ub. CPORT is a consensus of six interface predictors, and it deliberately overpredicts the interface, allowing HADDOCK to sample a large region of the surface [31]. Two additional controls were carried out, both using the LP/MS active residues on Rpn13, but with various passive residues on the ubiquitin side. First, instead of selecting only the CPORT predictions as passive residues on ubiquitin, the entire ubiquitin protein was made passive. This allows any location of ubiquitin to take part in the interaction in order to satisfy the experimental restraints, instead of just the residues predicted by CPORT. Table 3 (LP(all)) shows that removing thus the dependency on CPORT still gives acceptable results, but only as the fourth-best cluster instead of the top cluster as in the LP/MS run. Secondly, a control was performed where CPORT predictions were restricted to a conservative subset, taking only residues predicted by three interface predictors. This control run performed again worse than the LP/MS run, with the correct cluster now ranked as second (results not shown).

## Discussion

We have shown here that limited proteolysis/mass spectrometry (LP/MS) data, used with our MTMDAT-HADDOCK pipeline, is a valuable alternative to chemical shift perturbations (CSP) in the study of protein complexes. For the studied Rpn13-ubiquitin complex, LP/MS actually outperformed CSP, although both methods are likely to have similar outcomes on a larger set of model systems or even with the advantage on the CSP side. They also have similar properties concerning the nature of the obtained restraints, being not absolute distance restraints, but identifying only patches on the protein surface that are likely to be involved in intermolecular contacts. However, LP/MS is a superior method regarding the amount of time and sample used to acquire the data. High-resolution NOE-driven NMR-based structure calculations are time consuming and expensive in terms of preparation of isotopically labeled proteins, data recording and analysis, and furthermore require significant expertise. Backbone CSP data from NMR titration experiments, which are often used by structural biologists to alleviate the need for excessive structural analysis but which are sensitive to structural rearrangements, have the disadvantage of requiring a relatively stable and highly concentrated sample for backbone assignments, which are necessary even if crystal structures of the complex components are available. Approaches based on amino-acid selective labeling have been reported that do not require assignment [34], but these require rather expensive labeling of samples. In contrast, altogether 20 *μ*l of sample volume of both proteins with a concentration of 0.155 mM was needed for all LP/MS experiments performed in this study. The optimization and the actual time-course limited proteolysis/mass spectrometry experiments in triplicates can be performed within one working day, where the optimization should be limited to one range-finding experiment, where different protein:protease ratios are tested and in samples digested for 30 minutes. The choice of the best ratio is based on the presence of cleavage products. The full length protein should be present as well as shorter fragments. The subsequent time course experiments will then cover the full spectrum of fragment lengths from full length to shortest fragments. Peak assignment and data evaluation can be done within an additional day, whereas the time for the docking calculations varies and depends on the usage frequency of the HADDOCK web server. Usually, the docking is finished overnight, but can be as fast as one hour. If the user is registered at the eNMR platform for structural biology [35], the calculations can be even faster, despite the high number of structures needed in each iteration of this docking protocol. Thus, it is theoretically possible to get a structural model within four days with a backbone RMSD at the interface within ≈ 3 Å of the target structure.

A weakness of the LP/MS method is that it is limited by the proteases’ set of digested residues, whereas CSP is able to sample the entire residue space. Hence, there are probably many cases, where CSP would perform better than LP, but is more difficult to come by. However, this problem can be minimized by using different proteases. If only trypsin is used (lysines and arginines are the cleavage sites of trypsin), a maximum sequence coverage of around 12% can be achieved theoretically for an average protein [36]. If chymotrypsin (cleavage sites: tyrosine, tryptophan, phenylalanine, leucine, methionine) and V8 (Glu-C protease, cleavage sites: aspartatic acids, glutamic acids) are used additionally, the sequence coverage increases in average to 41%. This results in a high chance that at least one of these residues is part of the protein-protein binding interface and that it will be detected as an active residue. However, the smaller the interface, the higher the risk that no cleavage sites are present. In that case this method cannot be employed. This cannot be predicted but will be quickly detected by a lack of difference between the cleavage pattern of free and complexed components. Also, the user runs into risks of overinterpretation of results if only one or two active residues have been identified, which would be even worse if they are on different sites of the protein. In this case, further optimization is needed or another method needs to be employed. For example, in a recent study of an E2:E3 interface, limited proteolysis was employed to detect the binding interface on the E3 ligase site of TRIM21 [37]. Limited proteolysis managed only to detect one lysine to be involved in binding, altough other possible cleavage sites were present. This was largely due to the fact that binding affinity was observed to be very low in the high micromolar range. Thus, a difference between cleavage in the free and bound form is hard to discern. Although the Rpn13-ubiquitin complex presented a possible difficulty when it comes to proteolytic cleavage since ubiquitin was not cleaved by any of the proteases employed, we showed that LP/MS also works in this (rare) case of one protein being unsusceptible to proteolytic cleavage since this data can easily be complemented by predictions or data from other sources. In this study, interface predictions from CPORT were used successfully to complement the missing cleavage data. However, for Rpn13-ubiquitin, acceptable results could also be obtained by simply defining the entire missing protein (ubiquitin) as passive. Both of these runs also strongly outperformed docking based on CPORT predictions alone, showing that the experimental LP/MS data successfully drives the docking.

The detection of false positives is inherent to both the LP/MS and the CSP methods. A ligand binding event is often accompanied by allosteric effects such as conformational changes of the protein backbone at locations remote from the binding interface, which will be detected as chemical shift perturbations. This can also lead to a change in stability towards proteolytic cleavage, which will then be detected by the LP/MS method. However, the docking protocol developed here, with its filtering stage, appears to be robust against the detection of false positives. Interestingly, the positive control run (BL) and the CSP runs, where both input active residues are covering the reference more completely than the LP run perform worse in this case. We can only speculate that the interface of the case used here is difficult to dock, due to lack of secondary structure elements and involvement of mainly loops in the interface. Clearly, the user has to be careful while employing this method and do not trust results lightly if there are only few active residues identified. Although HADDOCK has been benchmarked and has been used to solve many important complex structures, it still relies on good input data and the usage of only one active residue will ultimately lead to failure of finding a trustworthy solution [20,38,39]. Also, although conformational change can give rise to false positives, it cannot be detected by this method in structural detail. Nevertheless, the tool presented here is very useful to the field of structural biology, since it combines limited proteolysis, mass spectrometry, and data-driven docking in a streamlined and unique way, and as shown, can produce structural models of reasonable quality.

The presented method should be especially well suited to samples resistant to crystallization and that interact in the intermediate exchange regime by NMR, such that NMR signals are broadened. Also, complexes with flexible regions are easily amenable for limited proteolysis. In this respect, intrinsically disordered proteins would be especially well-suited objects to study with MTMDAT. The interest in these unstructured proteins has increased due to their involvement in regulation and disease [40]. X-ray crystallography and small angle scattering fail here to contribute valuable structural information, since disordered proteins do not crystallize or do not form a stable measurable shape in solution. Despite recent advances in NMR spectroscopy and applications of the same on unstructured proteins [41-44], it is nevertheless a difficult undertaking to extract structural information from very often transient interactions within a disordered protein or a disordered protein and a binding partner. We speculate that MTMDAT can contribute to this field of structural biology by rapidly identifying regions of unstructured proteins that interact with their partners. However, the results will have to be interpreted with caution, since unstructured proteins are expected to undergo large conformational changes, leading to possible false-positive cleavage signals. In addition, obtaining reliable structural models of the complexes will most likely not be possible due to the large conformational changes involved.

In summary, these results show that MTMDAT-HADDOCK can be a tool to provide valuable structural insight in cases where classical NMR and X-ray crystallography are unfeasible, e.g. proteins that do not crystallize or have low solubility, or are too large for NMR spectroscopy. Also, protein production in large amounts and expensive labeling schemes can complicate or even prevent the structure determination. Despite recent advances in NMR methodology, protein-protein complex structure determination is usually not routinely done but needs manual inspection by experts. Therefore, in cases where high-throughput is desired, MTMDAT-HADDOCK can provide a solution, at the cost of atomic-level accuracy.

## Conclusions

In this article we have presented a new software tool, which evaluates limited proteolysis/mass spectrometry data quickly and extracts information regarding the residues involved in a particular protein-protein interaction. It provides directly the input file for data-driven docking on the HADDOCK web server to calculate a structural model of the complex. The MTMDAT-HADDOCK pipeline enables direct high-throughput modeling of protein complexes from mass spectrometry data, by providing an easy interface to obtain structural restraints for protein complex structure calculations. The usefulness of this approach has been validated successfully on the Rpn13-ubiquitin protein complex. Our results indicate that this approach is competitive, when compared to a similar approach using NMR-based chemical shift perturbation data alone. The level of expertise required to conduct the necessary experiments is however much lower than for NMR and sample requirements are much easier to fulfill. However, it should be viewed as an alternative approach, if sample requirements for NMR or crystallization cannot be fulfilled. As for structural models based on chemical shift perturbations, site-directed mutagenesis should be used to validate the model derived from our method.

## Methods

### Limited proteolysis, mass spectrometry, and data analysis

Both proteins, ubiquitin and Rpn13 were purified and stored in 20 mM NaPO_4_, 50 mM NaCl, 5 mM DTT, pH 6.5. Prior to proteolytic cleavage, they were diluted 1:10 resulting in a final concentration of 15.5 *μ*M. The optimal protease concentration for trypsin and V8 were determined in range-finding experiments [11]. For both enzymes, a protein:protease ratio of 50:1 was used. All proteolysis experiments were done in triplicates. In the time-course experiments (time points: 0, 1, 2, 5, 10, 20, 50, 100, 200 minutes) the reactions were stopped by adding 0.1% trifluoroacetic acid/50 % acetonitrile. A sample of each time point was mixed with *α*-cyanocinnamic acid matrix solution with a 1:1 ratio directly on the sample plate. Data acquisition was carried out as described previously [16]. The raw data was uploaded and evaluated as described (see above and [18]).

### Work flow

Rpn13 and ubiquitin were digested separately as well as in complex with a set of specific proteases as described above. In a stable protein complex, the proteolytic accessibility of cleavage sites in the interaction surface will be decreased, which is used to map the interacting residues. The use of several proteases results in higher sequence coverage and more accurate identification of the binding interface. If the proteins are large and there are many ambiguously assigned peaks it is helpful to digest the complex twice with different stoichiometries of the proteins involved. In this way, mass spectrometry peaks of the protein with the lower molar concentration can be suppressed. Mass spectra are evaluated with MTMDAT [18] to assign peaks and to generate 3D plot files, which consist of the relative cleavage propensity [16] at all cleaved sites and time points (file extension .csgnu). Data of the protein complex needs to be evaluated twice, once for each protein. By clicking a newly introduced button, called “H-DOCK” the user is prompted to upload the 3D plot files (.csgnu) of each protein and of both proteins in complex (altogether four files), and the PDB atom coordinates files of both proteins. A “docking preparation window” appears and MTMDAT displays the generation of ambiguous interaction restraints by comparing the relative cleavage propensities of the monomer with the complex. The user can provide MTMDAT with a threshold for picking interaction restraints to prevent overestimation of differences in relative cleavage propensities, which would result in false positives. This can be done iteratively in order to determine the best threshold. By clicking on the “H-DOCK” button in the docking preparation window, MTMDAT will write two files containing a list of ambiguous interaction restraints (AIRs) of both proteins and a HADDOCK (AIR) file (.tbl) for locally installed HADDOCK versions. Moreover, a HADDOCK parameter file is written, which includes all necessary parameters and data to perform the docking using the HADDOCK web server interface at http://haddock.science.uu.nl/services/HADDOCK. Furthermore, if data shows that residues are identified as being protected from proteolytic cleavage upon complex formation on more than one region, the program PATCHUP has been developed and included in the package, which identifies patches in an unbiased way. PATCHUP does a k-means clustering of the atom point cloud, then assigns each residue to the patch where most of its atoms are. It requires Biopython and Scipy. After patching, filter docking runs are performed by HADDOCK (one for each patch, with 50% random exclusion of active residues) to see which patch gives the best HADDOCK scores and clustering, before a final docking run is conducted, using active residues of the best interface patch with no random exclusion of active residues, and passive residues in immediate vicinity of active residues are also used as active residues. The cluster with best HADDOCK score should yield desired complex structures.

### Requirements and Improvements

MTMDAT comes as a software written in Java, relying on jre1.6.0 or later. The MTMDAT-HADDOCK pipeline was developed using the Spyder framework (http://www.spyderware.nl), which requires Python 2.6 or later. MTMDAT will work well on Unix and Windows operating systems provided you fulfil the requirements above. During the peak assignment a newly implemented undo-function increases the usability, since the misassignment or mistaken removal of peaks can be undone.

### Docking

MTMDAT-HADDOCK produced automatically the single input file for docking calculations on the HADDOCK web-server “file upload” interface (http://haddock.science.uu.nl/services/HADDOCK). As input structures for Rpn13 and ubiquitin, 2R2Y.pdb [25] and 1UBQ.pdb [45] were used, respectively. The interacting residues of ubiquitin identified by CPORT were used only as passive residues. In the CPORT control run, the AIRs were used as active. Passvie residues were identified automatically by HADDOCK. In the first run (filter), the default settings were used [21], except, that the number of structures calculated were increased from 1000 to 4000, 200 to 400, and 200 to 400, for the rigid body docking, semi-flexible simulated annealing, and water refinement, respectively, and all 400 structures were included into the analysis. These changes were used for all docking runs including the controls, where random exclusion of active residues was turned on. The resulting 400 structures in all runs were clustered using a cut-off of 7.5 Å, and a minimum cluster size of 4. The four lowest energy structures of each cluster were analyzed and fitted onto the reference complex [PDB:2Z59] using interface backbone atoms of residues within 10 Å from the binding interface using ProFit (http://www.bioinf.org.uk/software/profit/) for the iRMSD. For the lRMSD, the backbone atoms of the larger component of the complex were fitted on the reference, and the RMSD was calculated for the other component. The fraction of native contacts (fnat) was calculated by counting all contacts between the two proteins in the docked complex and dividing them by the number of all contacts in the reference structure (residue-wise). As a reference for RMSD calculations, the lowest energy structure of the ensemble in 2Z59.pdb has been used.

## Competing interests

The authors declare that they have no competing interests.

## Authors’ contributions

JH developed the algorithms of the MTMDAT part, took part in the pipelining between MTMDAT and HADDOCK, planned, performed, and evaluated the experiments, and wrote the manuscript. S. J. d. V. programmed the pipelining and took part in planning and evaluating the docking experiments and in writing the manuscript. LR produced and purified the proteins, KJW contributed to the writing of the manuscript. KDMH did most of the programming and took part in the pipelining. MS contributed to the writing of the manuscript. AMJJB took part in the planning and the evaluation of the experiments, and contributed to the writing of the manuscript. All authors read and approved the final manuscript

## Supplementary Material

Additional file 1 **MTMDAT-HADDOCK.** The MTMDAT-HADDOCK program package, including PATCHUP, packed as a .zip file. A manual how to use the software is included. Click here for file

## References

[B1] Engen J Wales T Hydrogen exchange mass spectrometry for the analysis of protein dynamics Mass Spectrom Rev 2006 25 158 170 10.1002/mas.20064 16208684

[B2] Barrera N Di Bartolo N Booth P Robinson C Micelles protect membrane complexes from solution to vacuum Science 2008 321 243 246 10.1126/science.1159292 18556516

[B3] Ruotolo B Benesch J Sandercock A Hyung S Robinson C Ion mobility-mass spectrometry analysis of large protein complexes Nat Protoc 2008 3 1139 1152 10.1038/nprot.2008.78 18600219

[B4] Young M Tang N Hempel J Oshiro C Taylor E Kuntz I Gibson B Dollinger D High throughput protein folding identification by using experimental constraints derived from intramolecular crosslinks and mass spectrometry Proc Natl Acad Sci USA 2000 97 5802 5806 10.1073/pnas.090099097 10811876 PMC18514

[B5] Back J de Jong L Muijsers A de Koster C Chemical cross-linking and mass spectrometry for protein structural modeling J Mol Biol 2003 331 303 313 10.1016/S0022-2836(03)00721-6 12888339

[B6] Sinz A Chemical cross-linking and mass spectrometry for mapping three-dimensional structures of proteins and protein complexes J Mass Spectrom 2003 38 1225 1237 10.1002/jms.559 14696200

[B7] Leitner A Walzthoeni T Kahraman A Herzog F Rinner O Beck M Aebersold R Probing native protein structures by chemical cross-linking, mass spectrometry, and bioinformatics Mol Cell Proteomics 2010 9 1634 1649 10.1074/mcp.R000001-MCP201 20360032 PMC2938055

[B8] Rappsilber J The beginning of a beatiful friendship: cross-linking/mass spectrometry and modelling of proteins and multi-protein complexes J Struct Biol 2011 173 530 540 10.1016/j.jsb.2010.10.014 21029779 PMC3043253

[B9] Maleknia S Downard K Radical approaches to probe protein structure, folding, and interactions by mass spectrometry Mass Spectrom Rev 2001 20 388 401 10.1002/mas.10013 11997945

[B10] Gerega S Downard K PROXIMO - a new docking algorithm to model protein complexes using data from radical probe mass spectrometry (RP-MS) Bioinformatics 2006 22 1702 1709 10.1093/bioinformatics/btl178 16679333

[B11] Carey J A systematic and general Proteolytic Method for defining structural and functional domains of proteins Methods Enzymol 2000 328 499 514 11075363 10.1016/s0076-6879(00)28415-2

[B12] Cohen S Ferre-D’amare A Burley S Chait B Probing the solution structure of the DNA-binding protein Max by a combination of proteolysis and mass spectrometry Protein Sci 1995 4 1088 1099 7549873 10.1002/pro.5560040607 PMC2143150

[B13] Kriwacki R Jiang W Siuzdak G Wright P Probing Protein/Protein interactions with mass spectrometry and isotopic labeling: analysis of the p21/Cdk2 complex J Amer Chem Soc 1996 118 5320 5321 10.1021/ja960752m

[B14] Lundqvist M Andrésen C Christensson S Johansson S Karlsson M Broo K Jonsson B Proteolytic cleavage reveals interaction patterns between silica nanoparticles and two variants of human carbonic anhydrase Langmuir 2005 21 25 11903 11909 10.1021/la050477u 16316131

[B15] Hennig J Bresell A Sandberg M Hennig K Wahren-Herlenius M Persson B Sunnerhagen M The fellowship of the RING: the RING-B-box Linker Region Interacts with the RING in TRIM21/Ro52, contains a native Autoantigenic Epitope in Sjögren Syndrome, and is an integral and conserved region in TRIM Proteins J Mol Biol 2008 377 431 449 10.1016/j.jmb.2008.01.005 18272178

[B16] Hennig J Ottosson L Andrésen C Horvath L Kuchroo V Broo K Wahren-Herlenius M Sunnerhagen M Structural organization and Zn2+-dependent subdomain interactions involving Autoantigenic Epitopes in the RING-B-box-Coiled-coil (RBCC) region of Ro52 J Biol Chem 2005 280 39 33250 33261 10.1074/jbc.M503066200 15964842

[B17] Wennerstrand P Dametto P Hennig J Klingstedt T Skoglund K Appell M Martensson LG Structural characteristics determine the cause of the low enzyme activity of two thiopurine S-methyltransferase allelic variants: a biophysical characterization of TPMT∗2 and TPMT∗5 Biochemistry 2012 51 5912 5920 10.1021/bi300377d 22747506

[B18] Hennig J Hennig K Sunnerhagen M MTMDAT: Automated analysis and visualization of mass spectrometry data for tertiary and quaternary structure probing of proteins Bioinformatics 2008 24 10 1310 1312 10.1093/bioinformatics/btn116 18388142 PMC2373922

[B19] Dominguez C Boelens R Bonvin A HADDOCK: A Protein-Protein docking approach based on Biochemical or Biophysical information J Amer Chem Soc 2003 125 1731 1737 10.1021/ja026939x 12580598

[B20] de Vries S van Dijk A Krzeminski M van Dijk M Thureau A Hsu V Wassenaar T Bonvin A HADDOCK versus HADDOCK: new features and performance of HADDOCK2.0 on the CAPRI targets Proteins 2007 69 726 733 10.1002/prot.21723 17803234

[B21] de Vries S van Dijk M Bonvin A The HADDOCK web server for data-driven biomolecular docking Nat Protoc 2010 5 883 897 10.1038/nprot.2010.32 20431534

[B22] de Vries S van Dijk A Bonvin A WHISCY: What information does surface conservation yield? Application to data-driven docking Proteins 2006 63 479 489 10.1002/prot.20842 16450362

[B23] de Vries S Bonvin A How proteins get in touch: interface prediction in the study of biomolecular complexes Curr Protein Pept Sci 2008 9 394 406 10.2174/138920308785132712 18691126

[B24] Husnjak K Elsasser S Zhang N Chen X Randles L Shi Y Hofmann K Walters K Finley D Dikic I Proteasome subunit Rpn13 is a novel ubiquitin receptor Nature 2008 453 481 488 10.1038/nature06926 18497817 PMC2839886

[B25] Schreiner P Chen X Husnjak K Randles L Zhang N Elsasser S Finley D Dikic I Walters K Groll M Ubiquitin docking at the proteasome through a novel pleckstrin-homology domain interaction Nature 2008 453 548 552 10.1038/nature06924 18497827 PMC2825158

[B26] Hamazaki J Iemura S Natsume T Yashiroda H Tanaka K Murata S A novel proteasome interacting protein recruits the deubiquitinating enzyme UCH37 to 26S proteasomes EMBO J 2006 25 4524 4536 10.1038/sj.emboj.7601338 16990800 PMC1589993

[B27] Yao T Song L Xu W DeMartino G Florens L Swanson S Washburn M Conaway R Conaway J Coher R Proteasome recruitment and activation of the Uch37 deubiquitinating enzyme by Adrm1 Nat Cell Biol 2006 8 994 1002 10.1038/ncb1460 16906146

[B28] Ito T Chiba T Ozawa R Yoshida M Hattori M Sakaki Y A comprehensive two-hybrid analysis to explore the yeast protein interactome Proc Natl Acad Sci USA 2001 98 4569 4574 10.1073/pnas.061034498 11283351 PMC31875

[B29] Gandhi T Zhong J Mathivanan SLK Chandrika K Mohan S Sharma S Pinkert S Nagaraju S Periaswamy B Mishra G Nandakumar K Shen B Deshpande N Nayak R Sarker M Boeke J Parmigiani G Schultz JJSB Pandey A Analysis of the human protein interactome and comparison with yeast, worm and fly interaction datasets Nat Genet 2006 38 285 293 10.1038/ng1747 16501559

[B30] Qiu X Ouyang S Li C Miao S Wang L Goldberg A hRpn13/ADRM1/GP110 is a novel proteasome subunit that binds the deubiquitinating enzyme, UCH37 EMBO J 2006 25 5742 5753 10.1038/sj.emboj.7601450 17139257 PMC1698896

[B31] de Vries S Bonvin A CPORT: A Consensus interface predictor and its performance in prediction-driven docking with HADDOCK Plos ONE 2011 6 e17695 10.1371/journal.pone.0017695 21464987 PMC3064578

[B32] DeLano W The PyMOL molecular graphics system 2002 San Carlos, CA, USA: DeLano Scientific

[B33] Janin J Henrick K Moult J Eyck L Sternberg M Vajda S Vakser I Wodak S CAPRI: a critical assessment of predicted interactions Proteins 2003 52 2 9 10.1002/prot.10381 12784359

[B34] Reese M Dötsch V Fast mapping of protein-protein interfaces by NMR spectroscopy J Am Chem Soc 2003 125 14250 14251 10.1021/ja037640x 14624553

[B35] Bonvin A Rosato A Wassenaar T The eNMR platform for structural biology J Struct Funct Genomics 2010 11 1 8 10.1007/s10969-010-9084-9 20229048 PMC2855812

[B36] Klapper M The independent distribution of amino acid near neighbor pairs into polypeptides Biochem Biophys Res Com 1977 78 1018 1024 10.1016/0006-291X(77)90523-X 911323

[B37] Espinosa A Hennig J Ambrosi A Anandapadmanaban M Sandberg Abelius M Sheng Y Nyberg F Arrowsmith C Sunnerhagen M Wahren-Herlenius M Anti-Ro52 autoantibodies from patients with Sjögren’s syndrome inhibit the Ro52 E3 ligase activity by blocking the E3/E2 interface J Biol Chem 2011 286 36478 36491 10.1074/jbc.M111.241786 21862588 PMC3196112

[B38] van Dijk M Bonvin A Pushing the limits of what is achievable in protein-DNA docking: benchmarking HADDOCK’s performance Nucleic Acids Res 2010 38 5634 5647 10.1093/nar/gkq222 20466807 PMC2943626

[B39] de Vries S Melquiond A Kastritis P Karaca E Bordogna A van Dijk M Rodrigues J Bonvin A Strengths and weaknesses of data-driven docking in critical assessment of prediction of interactions Proteins 2010 78 3242 3249 10.1002/prot.22814 20718048

[B40] Babu M vander Lee R de Groot NS Gsponer J Intrinsically disordered proteins: regulation and disease Curr Opin Struct Biol 2011 21 432 440 10.1016/j.sbi.2011.03.011 21514144

[B41] Schneider R Huang J Yao M Communie G Ozenne V Mollica L Salmon L Jensen M Blackledge M Towards a robust description of intrinsic protein disorder using nuclear magnetic resonance spectroscopy Mol Biosyst 2012 8 58 68 10.1039/c1mb05291h 21874206

[B42] Rezaei-Ghaleh N Blackledge M Zweckstetter M Intrinsically disordered proteins: from sequence and conformational properties toward druc discovery Chembiochem 2012 13 930 950 10.1002/cbic.201200093 22505141

[B43] Bibow S Ozenne V Biernat J Blackledge M Mandelkow E Zweckstetter M Structural impact of proline-directed pseudophosphorylation at AT8, AT100, and PHF1 epitopes on 441-residue tau J Am Chem Soc 2011 133 15842 15845 10.1021/ja205836j 21910444

[B44] Andresen C Helander S Lemak A Fares C Csizmok V Carlsson J Penn L Forman-Kay J Arrowsmith C Lundström P Sunnerhagen M Transient structure and dynamics in the disordered c-Myc transactivation domain affect Bin1 binding Nucleic Acids Res 2012 40 6353 6366 10.1093/nar/gks263 22457068 PMC3401448

[B45] Vijay-Kumar S Bugg C Cook W Structure of ubiquitin refined at 1.8 Åresolution J Mol Biol 1987 194 531 544 10.1016/0022-2836(87)90679-6 3041007

