# Supplementary material for: MTMDAT-HADDOCK: High-throughput, protein complex structure modeling based on limited proteolysis and mass spectrometry
Source: BMC Struct Biol. 2012 Nov 15;12:29. doi: 10.1186/1472-6807-12-29 (PMC3557227; doi:10.1186/1472-6807-12-29)
Supplement: Additional file 1 — MTMDAT-HADDOCK. The MTMDAT-HADDOCK program package, including PATCHUP, packed as a .zip file. A manual how to use the software is included. [file 1472-6807-12-29-S1.zip › MTMDAT-HADDOCK-2012/manual-mtmdat.pdf]

MTMDAT - A mass spectrometry data analysis tool for high-order structure probing of proteins and their interactions with HADDOCK pipeline

Klaus Hennig and Janosch Hennig

July 25, 2012

Contents

1 Introduction **1**

2 Installation and starting of MTMDAT **2**

2.1 MTMDAT-HADDOCK 2

2.1.1 Unix-based 2

2.1.2 Windows-based 2

3 Howto **3**

3.1 Starting 3

3.2 The target sequence 3

3.3 The mass spectrometry file upload 6

3.4 Peak picking 6

3.4.1 Automatic noise filter ON 6

3.4.2 Automatic noise filter OFF 8

3.5 Peak assignment 8

3.6 Creating the plot-files 10

3.7 The 3D Visualisation 11

3.8 The MTMDAT-HADDOCK pipeline 12

3.9 Other features 15

List of Figures

1 MTMDAT - GUI 4

2 MTMDAT - Right top corner 4

3 MTMDAT - Virtual Cleavage 5

4 MTMDAT - Parameter window 5

5 MTMDAT - MS file display 7

6 MTMDAT - Peak picking 7

7 MTMDAT - Peak assignment 9

8 MTMDAT - Remove peaks from assignment list 9

9 MTMDAT - Save plot 10
10 Time-course profile 11
11 3D visualisation 13
12 HADDOCK docking preparation 15

1 Introduction

MTMDAT was mainly developed to evaluate MALDI-TOF-MS spectrometry data of limited proteolysis experiments for high-order structure probing of proteins. To understand the principles of this experiment one should read the references given in the bibliography [1, 2, 3]. This manual focuses therefore on explaining how to process your data with MTMDAT on a practical level. However, other applications of MTMDAT are possible, e.g. in peptide synthesis and further development will spreaden the applicability of MTMDAT.

In the following chapters we will explain how to use MTMDAT in a most efficient way to get your evaluation done in a matter of hours instead of days or weeks in case you do not have this program at hand. To do this we will guide you through the entire process by using the example provided with the distribution of the program. Therefore it is important that you have subfolders called seq, plots, mdata, and assignment-lists in the folder where the executable script is located. In there, example files should be present. This example is based on published data of a RING-RBL region of Ro52 [4]. It has been cleaved with three different enzymes, namely trypsin, chymotrypsin and V8 (Glu C protease).

If you follow the instructions you will have no problem to do likewise with your own data.

Now in the new version with the MTMDAT-HADDOCK pipeline we do the instruction in the same way by providing example files of two proteins supposed to form a complex. We call these proteins proteinA and proteinB.

Good Luck, Hennig J., and Hennig K.

2 Installation and starting of MTMDAT

2.1 MTMDAT-HADDOCK

MTMDAT together with the HADDOCK pipeline should work on all operating systems provided you have Java Runtime Environment jre1.6.0 or later installed and python 2.6. For unix systems you have downloaded a tar file (MTMDAT-HADDOCK.tgz) and for a windows system a zip file (MTMDAT-HADDOCK.zip).

2.1.1 Unix-based

Put the MTMDAT-HADDOCK.tgz file in a folder of your choice. Type tar -xzf MTMDAT-HADDOCK.tgz in the command line to unpack the tar file. A folder should appear named "MTMDAT-HADDOCK-2012". Go to that folder and run ./mtmdat from the command line.

2.1.2 Windows-based

You have to unpack the downloaded zip file manually and start MTMDAT by clicking on the file mtmdatwin.bat.

3 Howto

It is always recommended to have a printout of the spectra you want to evaluate before you start. These are not provided for the example.

3.1 Starting

After installation, check if the following folders and files are present, which are needed to go through this instruction:

- seq: contains a file called RING-RBL.seq, where you can find the sequence of the target protein in one-letter-code
- mdata: contains the raw data of mass spectrometry spectra of the RING-RBL protein construct digested with trypsin with different time points (1, 2, 5, 10, 20, 50, 100, and 200 minutes).
- plots: contains the resulting plot files

After starting the program (see section 2), the graphical user interface will appear (see fig.1)

3.2 The target sequence

In the right top corner of the main window you see four rows (see fig.2). The sequence, enzyme, MS data file, and the date. In the sequence field you can upload a sequence file or paste in the query sequence. The one-letter-code is required, but characters not assigned to any of the 20 native amino acids are just filtered out. Following the example you can type into the sequence field the path of the file RING-RBL.seq (./seq/RING-RBL.seq).

The second field is a pull-down menu, where you can choose the enzyme you cleaved your protein with. Start in our case with trypsin.

Below these four fields you see two browser buttons (red arrow - backward, and green arrow- forward). By clicking on the forward button once, the program performs a virtual cleavage of your protein with the enzyme trypsin and lists the resulting fragments in the lower left window according to (see fig.3):

1. column: number of appearance and sorted after mass

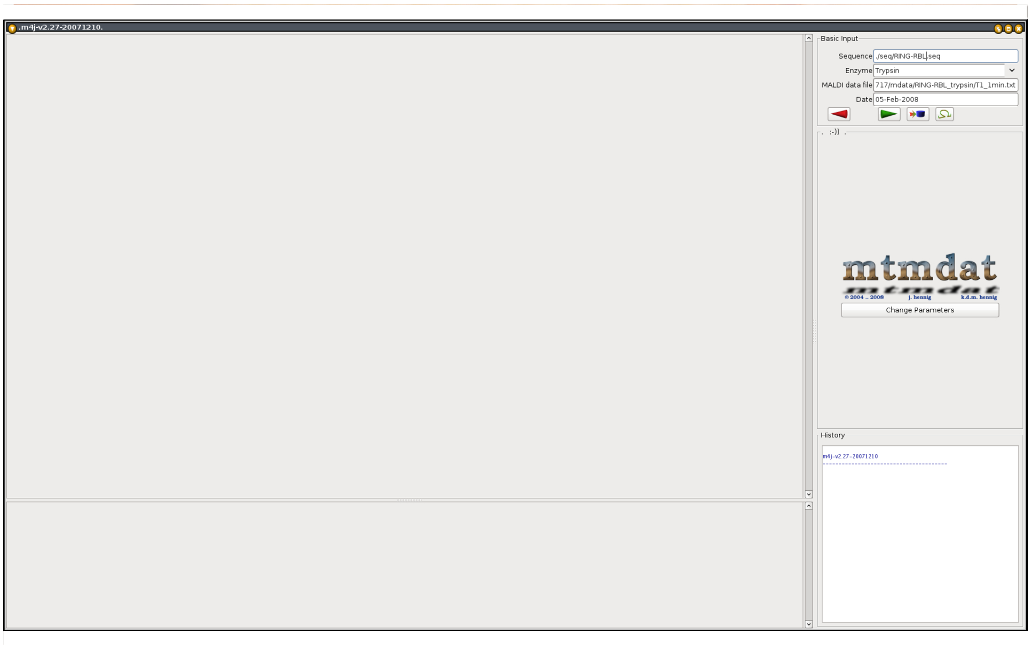

Figure 1: Graphics User Interface of MTMDAT. The main window

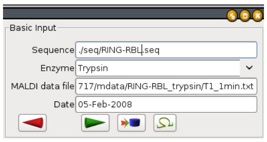

Figure 2: The right top corner of the main window, where you can choose you target sequence to be digested by an enzyme of your choice. The mass spectrometry file you choose in the third row

2. column: number of residues in the fragment
3. column: position of the fragment within the protein sequence
4. column: average mass
5. column: monoisotopic mass
6. column: number of missed cleavage sites

Now go back a step (the fragment list will vanish and a zero appears in between the browser buttons) and choose in the menu bar "Edit"-"change parameters". A parameter window will appear (see fig.4). Some radio buttons are already turned on. If you click on "Show sequence" and click on the

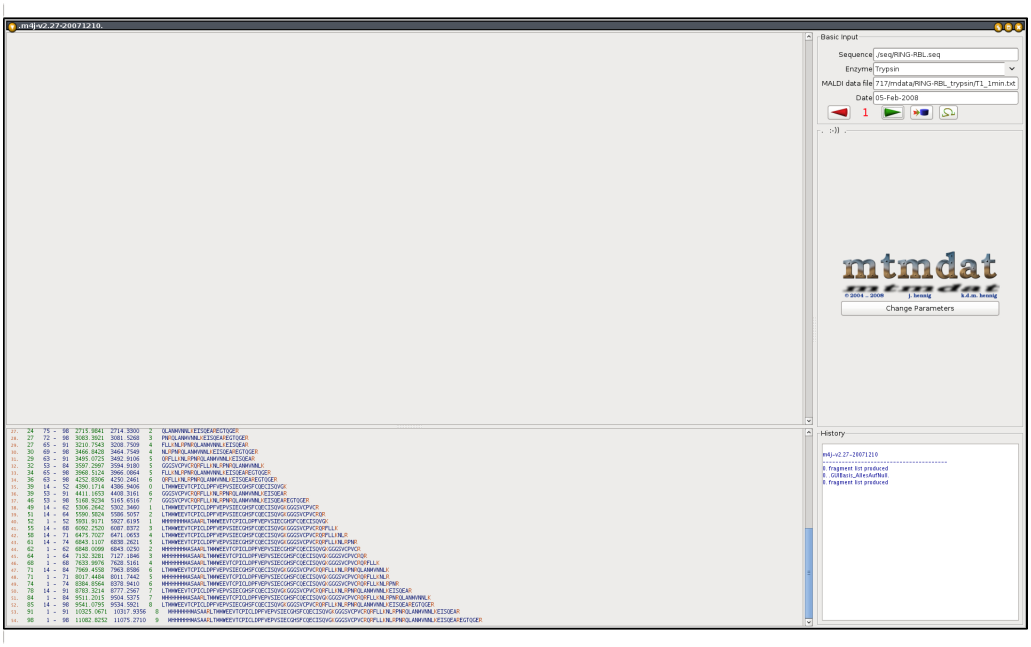

Figure 3: All virtual cleavage products are listed in the lower left frame of MTMDAT's main window.

forward button, the fragment list is displayed again with a seventh column, showing the sequence of each fragment and highlighting the cleavage sites in red (see fig.3).

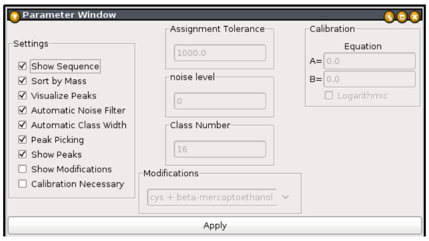

Figure 4: MTMDAT's parameter window where you can select and deselect certain features such as the automatic noise filter, calibration and so on.

When turning "Show modifications" on in the parameter window you can choose some modifications (e.g. met oxidized) and these modified masses will be as well calculated and listed.

3.3 The mass spectrometry file upload

Now let's start evaluating the first MS spectrum. To upload the first MS spectrum file you can whether type in the file location and name in the appropriate field (right top corner of main window, behind MS data file, see fig.2). Or leave it empty and press the forward button directly, which you would have to do anyways. Then a window will appear where you can choose the file you wish to upload. Now choose from the "mdata" folder "RING-RBL-trypsin", and upload the file called "T1-1min.txt". In the upper left window some file information will be displayed (see fig.5):

1. File header.
2. Followed by some statistical evaluation. The class width depends on the class number you choose in the parameter window. It divides the maximal intensity into equal parts and then calculates how many peaks are present in each part. This gives clues if you want to choose a noise level cut-off manually.
3. A similar approach as the class width approach follows afterwards, where an intensity mean value is calculated. The program calculates then how many peaks have higher intensity than the mean intensity or its multipliers.

3.4 Peak picking

Here you have two different approaches to choose from. In the parameter window you can toggle the automatic noise filter on and off. For a spectrum with good signal-to-noise it is recommended to use the automatic noise filter since it will increase the speed of evaluation even more. On the other hand it can give problems when the baseline is not horizontal or if peaks are obscured by noise. Then it might be better to turn the automatic noise filter off.

3.4.1 Automatic noise filter ON

Make sure that the automatic noise filter is turned on in the parameter window. Then it is enough to click on the forward button a third time (the number 3 should appear between the browser buttons). A new statistical analysis will be displayed and the noise level is calculated and shown (in case of our example it is 2327.17. By clicking on the browser button a fourth time the program will pick the peaks and display them schematically with their corresponding mass and intensity (see fig.6).

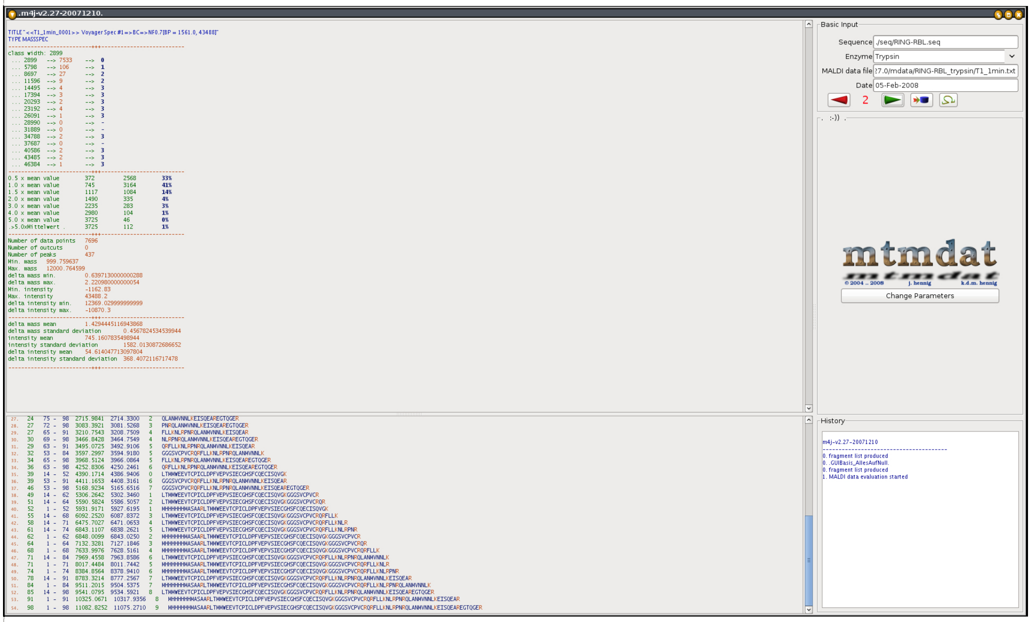

Figure 5: The left top frame displays the mass spectrometry file properties.

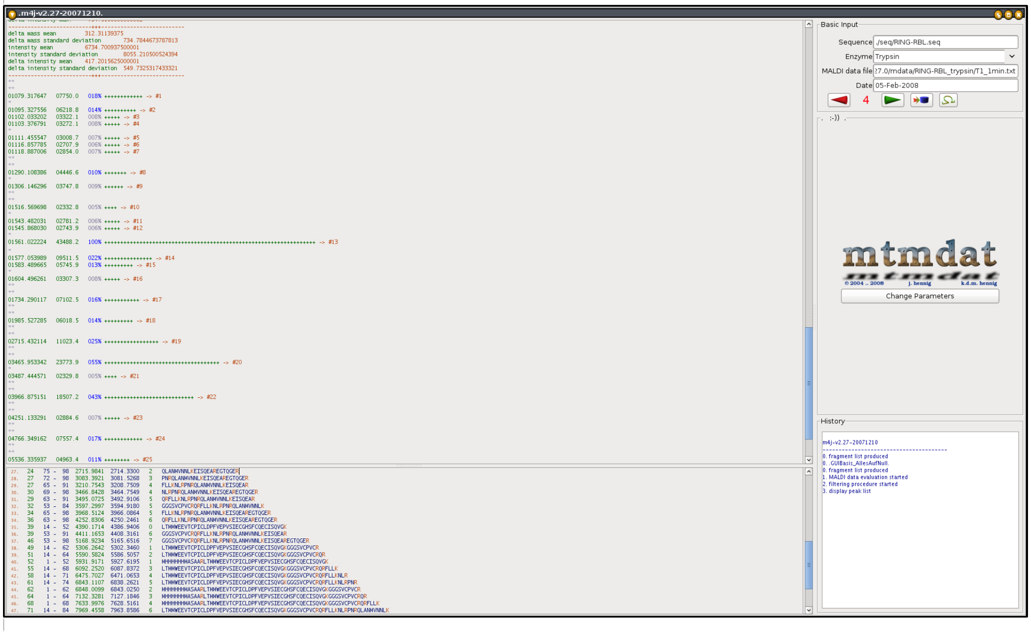

Figure 6: After noise filtering the peaks are picked and siplayed in the left top frame of MTMDAT's main window.

3.4.2 Automatic noise filter OFF

First, click three times on the backwards button (a "1" should appear between the browser buttons) and click on the radio button automatic noise filter in the parameter window to turn it off. No you are able to write a number in the field "noise level" in the parameter window. Let us try 3000 first. Click on "apply", go back to the main window and click on the forward button three times (until a "4" appears). Now the noise level is displayed again and it shows our manually chosen 3000. You will realize that fewer peaks are picked. For example the full-length RING-RBL peak (m/z 11082) is not picked anymore.

As mentioned in the beginning of this chapter it is wise to have a printout of the original spectrum in front of you to make sure all peaks you want to have picked are in your peak list.

3.5 Peak assignment

We continue with the picked peaks without automatic noise filter and press the forward button a last time (a "5" should appear). A new window will appear with the title "Peak Assignment Window" (see fig.7). Here all peaks are listed together with their possible assignment candidates. On top of the list the number of peaks (20), the noise level (3000), the mass tolerance (0.0010 or 1000 ppm), and the mean intensity (9088) is shown. The mass tolerance of 1000 ppm is the default value and can be changed in the parameter window by clicking on the radio button for "change mass tolerance". Then you are able to type into the "mass tolerance" field to the right the ppm value you desire (in case you haven't calibrated your spectrometer very well).

As mentioned, in our case we have 20 peaks. Let us look at peak number 20. It has a m/z of 9533.22 and its intensity is 5040. To the right of these values the relative peak intensity is given with 2.77 %. The next two lines show to which peptide fragment this peak has been assigned to. In our case it is a peptide corresponding to residues 14-98 of the query sequence. To the right of that you see the deviation to the actual mass, which is here -7.9 and further to the right the expected mass (9541.08) is shown. Unmodified means here that no chemical modifications of amino acids are part of the fragment. The number of charges is displayed as well. The last row shows the amino acid sequence of the peptide fragment.

As you can see in our example there are some peaks not assigned and the assignment of some is unlikely (e.g. peak 1, which is the six-times charged

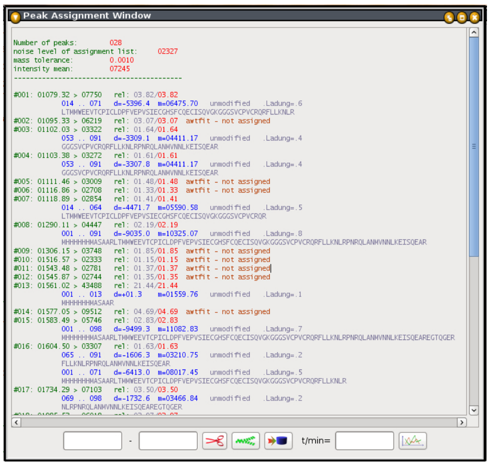

Figure 7: Picked peaks are listed in the peak assignment window with their assignment candidates.

peak of fragment 14-71, although the 1-5 charged peaks are not visible). These peaks you want to remove from the assignment list. This you do by simply writing the peak number into the lower left field. For example we want to remove the peaks 1-7, since there assignment is wrong or they don't have any assignment. Do as shown in fig.8 and write in the lower left field 1 and in the field to right of it 7. Then you press the button which depicts scissors.

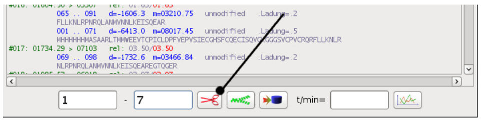

Figure 8: As described you can write the peak numbers in the appropriate fields and remove them by clicking on the scissors button.

Now only 14 peaks are left and the relative intensities for each are recalculated. Remove now peak 1, 2-4 and 13 of the current list since these are not assigned or wrongly assigned as well. We have 9 peaks left. You can combine single charged and double charged species of the same fragment by writing in our case 2 in the lower left field and 5 in the next field (same as before) but press the button depicting a zipper instead. Only 8 peaks are left, but the relative intensity of peak 2 has been added to peak 5 (which is now peak 4). The same can be done with 2 and 5, and 6 and 8 of the current list.

These steps are reasonable, since both peaks are related to the same fragment

in your reaction tube. So in the end you have only 6 peaks left. The word "Sum" in the end of the line indicates that this peak results from adding up peaks of the same fragment but different charge numbers.

You can save the peak assignment list for book-keeping purposes by clicking on the button to the right of the zipper-button (save peak list). You might want to save it under the folder assignment-lists in a new folder. In that way you don't mess up the example files.

As of 2009 (Version 2.30.5) you have a very useful undo function during peak assignment. Just use the key combination ctrl-z for undoing.

3.6 Creating the plot-files

The crucial point arrives now. You opened in our case the spectrum of your protein which has been cleaved for 1 minute in a trypsin solution. In the lower right field of the assignment window you should write the cleavage time in minutes (1) and then press the button to the right of it (see fig.9).

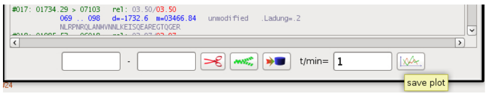

Figure 9: You can create a plot file by writing the time point into the appropriate field (here a "1" for one minute) and click on the save plot button to save it.

Choose a proper location where you want to save it and choose a proper name. Now look into that folder. You should see six new files. They all start with the prefix you have chosen as a filename when you saved it. And they all end with the fragment identifier (location of the fragment in your query sequence). If you open such a file in a text-editor of your choice you will see a dublet consisting of the time point (1 min) and the relative intensity of the corresponding peak.

The same can now be done with all subsequent time points. When you save the assignment for the next time, the dublet will automatically be written into the same files for each fragment provided you have chosen the same prefix. You can check one of them in the example files provided. Open i.e. the file T114-52 in the RING-RBL/trypsin folder. There you see seven dublets of the time points 2, 5, 10, 20, 50, 100, and 200 with their corresponding relative intensities of fragment 14-52. There was obviously no peak corresponding to that fragment in the spectrum for time point 1 minute.

The generated plot-files can now be used in any plotting program to produce time-course profiles in order to observe the degradation of unstable or accumulation of stable peptide fragments in your cleavage solution. An example of such a graph plotted with Gnuplot is shown in fig.10.

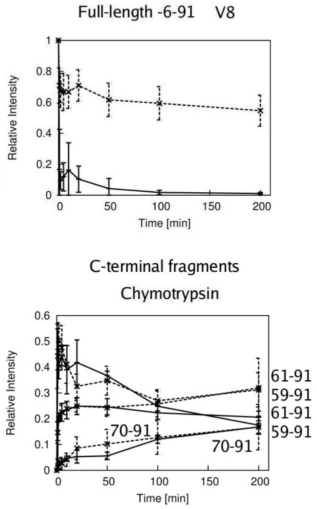

Figure 10: Typical time-course profile generated with Gnuplot from MTM-DAT plot-files to observe the degradation of unstable and accumulation of stable fragments.

3.7 The 3D Visualisation

When you evaluated all your time points and you have your plot-files ready you are only one click away from getting a file for visualising your data in three dimensions. Go back to the main window and click on the button depicting an omega. You will be asked to pick one of the plot-files of your current evaluation (choose an arbitrary one). Then a window pops up asking for the common prefix of all the files you want to include (in our case "T1"). The next window asks for the time points you want to include, showing already a default with all time points of your evaluation (you might want to remove some).

After that you have to choose a location and a file name where the 3D plot-files should be saved. You will be rewarded with the generation of 11 plot-files (containing triplets: residue identifier or similar, time, and relative cleavage propensity):

1. "filename".csgnu: this plot-file is ready-to-use for gnuplots 3D plotting procedures with only cleaved cleavage sites
2. "filename".csgnux: same as the previous but including all cleavage sites, even those which haven't been cleaved (relative cleavage propensity is 0)
3. "filename".csgnuxx: same as the previous, but instead of having a residue identifier each site gets numbered from 1 to 10 in our example (this file is only generated when you pressed the "save all" button in the main window once during your evaluation. An "filename".info file will be generated which should be located in the same folder as your 3D-files)
4. "filename".cslin: same as .csgnu but instead of the time you will have linear numbering of your time points in case you want to plot your time axis linearly
5. "filename".cslinx: a combination of .csgnux and .cslin
6. "filename".csnom: this is not a plot file but can be used to search more easily for possible mistakes in your evaluation
7. "filename".cssum: same as previous
8. "filename".cstab: a plot-file for spreadsheet programs
9. "filename".liste: another file to search for possible mistakes
10. "filename".tabka: same as .cstab but instead of relative cleavage propensity of each cleavage site you have the relative intensities of each fragment listed
11. "filename".tripl: combination of the .csgnu file types and .tabka values

These files can be used to plot figures like you see in fig.11. Now you are basically finished with your evaluation.

3.8 The MTMDAT-HADDOCK pipeline

This is a very useful implementation in case you have e.g. two proteins (proteinA and proteinB) with known structure (PDB files are available) and you would like to determine their complex structure, using HADDOCK [5].

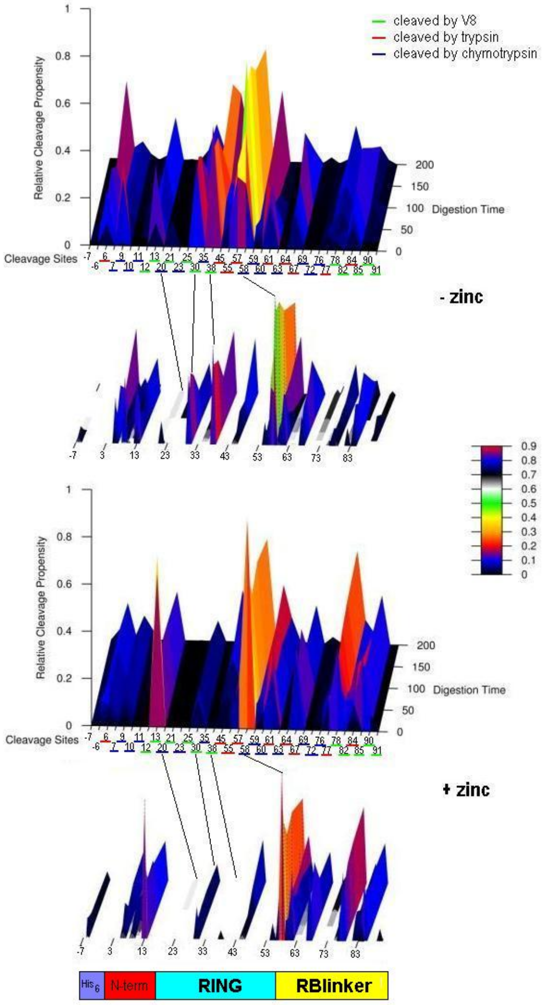

Figure 11: A 3D visualisation of limited proteolysis data generated by Gnuplot using MTMDAT's 3D plot files.

Therefore, you have to do a proteolysis experiment of proteinA, proteinB and both proteins together. You evaluate the experiments as described above until you get the 3D files ("filename".csgnu are needed for further steps). Important to note here is that you have of course to evaluate the experiment of the complex twice (proteinA-proteinB, where only peaks of protein A are taken into account, and then proteinB-proteinA, where only peaks of protein B are taken into account). In the version you downloaded, you will find a folder called "4haddock". Here are several example files located to test this feature. You have four .csgnu files (RT.csgnu from protein A, RUT.csgnu for

the complex AB, but evaluated is the cleavage pattern of protein A, UT.csgnu for protein B and URT.csgnu for the complex, but protein B's cleavage pattern is evaluated). Furthermore, you have the structure coordinate files of both proteins (protein A: 2R2Y.pdb and protein B: 1UBQ.pdb). Now you simply click on the "H-DOCK" button in the main window. MTMDAT asks you now for the location of the .csgnu file for proteinA, followed by proteinAproteinB, then proteinB and proteinBproteinA. Afterwards it asks for the location of the PDB file for proteinA and proteinB. Now it wants you to choose a certain threshold value between 0-100 %. Here it is recommendable to look at a plot of your 3D files or to look at the values in these plotfiles. The difference between the relative cleavage propensity of different residues should not be overestimated. For instance if residue K81 of proteinA in absence of proteinB shows a relative cleavage propensity of 1 and in presence of proteinB 0.1, then it probably interacts with proteinB and can be chosen by MTMDAT as an active AIR (ambiguous interaction restraint). Here you could even choose a threshold of 90 %. At this point it is not possible to predict a certain threshold where you can be sure that you do not pick false positives. This depends as well on the number of cleaved cleavage sites and thus among other things on the size of the protein.

After this a new window appears called "Docking preparation window (Finding Ambiguous Interaction Restraints)" (fig.12). The calculation of AIRs based on relative cleavage propensities is documented here.

You will be able to see a couple of tables. The last two are the most interesting ones, where the final AIRs are listed. In the first column the residue number is given and corresponds to the position of the residue in the input sequence during evaluation of your proteolysis data. The second column provides the mean value of relative cleavage propensity over all time points for this residue. The n-value in the third column says in how many time points this residue has been cleaved, followed by the fourth column, where a ratio between n and the total number of time points is given. The fifth column ($\Delta\%$) is the most interesting, since this value gives the difference in relative cleavage propensity of the corresponding residue between the monomer and the complex. This is as well a useful information to pick the right threshold. You just have to repeat the procedure and adjust the threshold accordingly, which is done in a matter of seconds. The remaining columns show the corrected relative cleavage propensities for each time point.

In the following the active (primary) AIRs of proteinA and proteinB are listed. For the locally installed HADDOCK versions, where a separate AIR file (.tbl) is necessary, passive (secondary) AIRs are automatically picked to be the sequence neighbors of active AIRs. Since some of them might not be

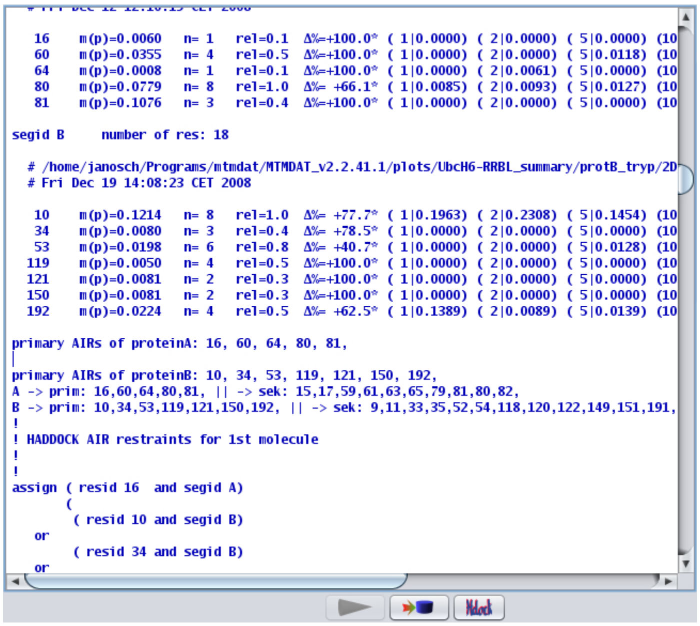

Figure 12: The Docking preparation window, where active ambiguous interaction restraints are calculated based on the relative cleavage propensities of both proteins involved. The HADDOCK parameter file is generated here.

passive AIRs it is good to double check by looking at the structure of the proteins.

However, if you now press the H-DOCK button in this docking preparation window, the afore mentioned .tbl file for locally installed HADDOCK versions is generated and written into the folder where the .csgnu files (3D plot files) of proteinA are taken from. Into the same folder two text files are saved (listeA.txt and listeB.txt), containing a list of AIRs for proteinA and proteinB respectively. In the same location the HADDOCK parameter file is written (called "bienlein2haddock.web"). This file contains all the information you need to start a docking on the HADDOCK web server (http://haddock.chem.uu.nl) in the file upload interface. Even passive AIRs are automatically added based on the proximity to the active residues and their surface availability.

3.9 Other features

As you might have noticed, there is a radio button in the parameter window named "calibration necessary" which has not been mentioned yet. This is in

case that you address a broad mass range and if you want to compensate the lower relative intensities of bigger fragments compared to smaller fragments as is described in [3].

References

[1] J. Carey. A systematic, general proteolytic method for defining structural and functional domains of proteins. *Meth Enzymol*, 1999.

[2] S.L. Cohen, A.R. Ferre-D'amare, S.K. Burley, and B.T. Chait. Probing the solution structure of the dna-binding protein max by a combination of proteolysis and mass spectrometry. *Prot Sci*, 4:1088–1099, 1995.

[3] J. Hennig, L. Ottosson, C. Andrésen, L. Horvath, V.K. Kuchroo, K. Broo, M. Wahren-Herlenius, and M. Sunnerhagen. Structural organization and zn^{2+}-dependent subdomain interactions involving autoantigenic epitopes in the ring-b-box-coiled-coil (rbcc) region of ro52. *J Biol Chem*, 280(39):33250–33261, 2005.

[4] J. Hennig, A. Bresell, M. Sandberg, K.D.M. Hennig, M. Wahren-Herlenius, B. Persson, and M. Sunnerhagen. The fellowship of the ring: The ring-b-box linker region interacts with the ring in trim21/ro52, contains a native autoantigenic epitope in sjögren syndrome, and is an integral and conserved region in trim proteins. *J Mol Biol*, doi:10.1016/j.jmb.2008.01.005, 2008.

[5] C. Dominguez, R. Boelens, and A.M.J.J. Bonvin. Haddock: A protein-protein docking approach based on biochemical or biophysical information. *JACS*, 125:1731–1737, 2003.
